# Investigation of Antistress and Antidepressant Activities of Synthetic Curcumin Analogues: Behavioral and Biomarker Approach

**DOI:** 10.3390/biomedicines10102385

**Published:** 2022-09-24

**Authors:** Haya Hussain, Shujaat Ahmad, Syed Wadood Ali Shah, Abid Ullah, Mazen Almehmadi, Osama Abdulaziz, Mamdouh Allahyani, Ahad Amer Alsaiari, Mustafa Halawi, Edrous Alamer

**Affiliations:** 1 Department of Pharmacy, Shaheed Benazir Bhutto University Sheringal Dir (Upper), Dir 18000, Pakhtunkhwa, Pakistan; 2 Department of Pharmacy, University of Malakand Dir (Lower) at Chakdara, Chakdara 18800, Pakhtunkhwa, Pakistan; 3 Department of Clinical Laboratory Sciences, College of Applied Medical Sciences, Taif University, Taif 21944, Saudi Arabia; 4 Department of Medical Laboratory Technology, College of Applied Medical Sciences, Jazan University, Jazan 45142, Saudi Arabia; 5 Medical Research Center, Emerging and Epidemic Infectious Diseases Research Unit, Jazan University, Jazan 45142, Saudi Arabia

**Keywords:** stress, depression, curcumin analogs, oxidative stress, antioxidant, antidepressants, in vivo study, antistress, biomarkers, hippocampus

## Abstract

Depression is a serious psychiatric disorder that affects millions of individuals all over the world, thus demanding special attention from researchers in order to investigate its effective remedies. Curcumin, along with its synthetic derivatives, is recognized for its incredible pharmacological activities. In this study, methyl, methoxy and chloro-substituent synthetic curcumin analogues **C1**–**C3** were respectively tested for free radical-scavenging activity. Behavioral studies were performed using chemical-induced and swimming endurance tests as stress models, and forced swim tests (FSTs) and tail suspension tests (TSTs) as depression mice models. Biochemical examinations were performed after a scopolamine-induced stress model by decapitating the mice, and brain tissues were isolated for biochemical assessment of catalase (CAT), superoxide dismutase (SOD), glutathione (GSH), and malondialdehyde (MDA). The curcumin analogue **C2** exhibited higher DPPH (2,2-diphenyl-1-picrylhydrazyl) and ABTS (2,2′-azinobis-3-ethylbenzothiazo-line-6-sulphonate) free radical-scavenging potential, having IC_50_ values of 45.18 µg/mL and 62.31 µg/mL, respectively, in comparison with reference curcumin and tocopherol. In the chemical-induced test, **C2** (80.17%), **C3** (72.79%) and **C1** (51.85%) revealed higher antistress responses by significantly reducing the number of writhes, whereas the immobility time was significantly reduced by **C2** and **C3** in the swimming endurance test, indicating excellent antistress potential. Similarly, **C2** and **C3** significantly reduced the immobility times in FST and TST, demonstrating their antidepressant properties. The biomarkers study revealed that these compounds significantly enhanced hippocampus CAT, SOD and GSH, and reduced MDA levels in the scopolamine-induced stress mice model. These findings suggest the potential of curcumin analogues (**C2** and **C3**) as antistress and antidepressant agents.

## 1. Introduction

Depression is considered among the most common forms of mental disorders, associated with many manifestations that affect the quality of an individual’s life [1]. When a person becomes frustrated, angry or nervous, a special type of feeling or condition occurs in the human body. Such feelings or conditions are known as stress. In reality, stress is the body’s response to demands. As a result of these responses, the body undergoes the synthesis of various chemical compounds, referred to collectively as stressors [2]. Stress is defined as the disruption of the normal physical and psychological balance in a person as a result of exposure to events that cause pressure and tension, and the undesired events are referred to as stressors [3]. Depressive episodes are associated with negative life experiences [4], and stressful life events are linked to the genesis of depression [5]. Psychological stress, especially depression, is a risk factor for dementia, a therapeutic target for depression psychotherapy, and a prognostic biomarker for strokes and dementia [6]. At present, depression has gained the considerable attention of researchers due to its negative impacts on the mind and body [7].

The exact cause of the disease is still unknown to researchers. However, a number of stressors are believed to be the etiological factors associated with depression, and neuroinflammation plays an important role in the onset of both neurodegenerative diseases and depression [8,9]. A number of pro-inflammatory markers, including tissue necrosis factor alpha (TNF-α), C-reactive protein (CRP), interleukin-6 (IL-6), and interleukin-8 (IL-8) of both blood and cerebrospinal fluid, were elevated in mental disorders such as depression, schizophrenia and anxiety [10]; multiple factors including psychological influences, both genetic and biological, were also involved in the initiation of depressive episodes [11]. Facial emotional expressions are among the contextual factors that can modulate brain activations and help in the understanding of the manners of social cognition linked with numerous neurological and psychiatric disorders [12]. The roles of the neural basis in the central nervous system during fear and stress are obvious from history. However, there is growing evidence of the role of the autonomic nervous system in stress and fear, providing the functional interplay between brain- and heart-related underlying processes in stress and fear conditioning [13].

Another vital contender amongst the etiological factors leading to depression is oxidative stress [14]. Due to high oxygen and nutrition demands, the brain is more vulnerable to oxidative stress in comparison to other organs [15]. The correlation of oxidative stress with the severity of depression is indicated by various preclinical and clinical studies [14,16,17]. Oxidative stress is mainly responsible for the impact of ageing, and is linked to depression [16,17,18]. When the concentration of reactive oxygen species (ROS) in cells exceeds the antioxidant level, oxidative stress occurs, which causes neuronal degeneration under extreme conditions [19]. In the biological system, oxidative stress indicates an imbalance between the quenching and production of oxygen free radicals [20]. According to research, depression is caused by oxidative stress dysregulation, which include imbalances in free radicals, antioxidants, oxidative damage, total antioxidant capacity, and autoimmune response products [14,21]. Stressful conditions are generally connected with the stimulation of many cellular activities, which raise the body’s energy requirements and thus increase the oxygen free radicals, resulting in oxidative stress [22]. In unipolar depression, oxidative stress can raise MDA levels while decreasing antioxidant levels [23]. Oxidative stress is persistently responsible for damage, producing harmful effects on mitochondria, causing mitochondrial malfunctions and slowing tissue regeneration. Reactive oxygen species play an important role in neurodegeneration in aged individuals and rodents [24]. Psychological, social, and physical stressors could be assessed in experimental animal models [25,26].

In the body, a complex system comprising enzymes and antioxidant metabolites protects the important cell components from oxidative damage by inhibiting their generation or removing these reactive species [27]. The antioxidant system consists of many enzymes, such as catalase (CAT), superoxide dismutase (SOD), glutathione (GSH) and the product of lipid peroxidation malondialdehyde (MDA), which play a pivotal role in oxidative stress. [28]. Antioxidants scavenge free radicals, protecting humans from oxidative stress [29], and act as reducing agents, oxidizing themselves by slowing the oxidation process; they include ascorbic acid and polyphenols [27]. Living organisms survive under stressful conditions by undergoing biochemical, morphological, and physiological changes. These changes reduce demands and help regulate behavior via a variety of behavioral responses [30]. As a result, maintaining a balance between oxidant and antioxidant concentrations is essential for optimal physiological processes [31]. Hence, treatment with antioxidants has been revealed to improve psychiatric symptoms in clinical trials [32].

Similarly, several substances qualify as antioxidants, including carotenoids, tannins, catechins, polyphenolics, and gallic acid derivatives. Ascorbic acid and tocopherol have the most effective antioxidant profiles, protecting against a number of diseases such as heart disease and cancer. As a result, researchers are focusing on the finding and development of effective, less expensive, and safer medicines to treat stress-related ailments. In addition, anxiolytics, benzodiazepines, and CNS stimulants (caffeine and amphetamine) have been reported as stress-reducers, but they are linked with adverse effects, physical dependence, tolerance, and toxicity when taken for a long time, restricting their clinical use [2,33].

Curcumin is a polyphenol that is naturally found in the rhizome of *Curcuma longa* L., [34], and has a wide spectrum of biological activities, including antioxidant, neuroprotective, anti-inflammatory, anticancer, hypoglycemic, antimicrobial and antiviral, and it is also used as a dietary pigment and spice [35,36,37,38,39]. Curcumin’s antidepressant potential has been shown in a number of preclinical studies in animal models using mice and rats [40,41,42]. Besides the tremendous biological properties of natural curcumin, their synthetic analogues have also been reported in various studies to exhibit antioxidant, cytotoxic, neuroprotective, anti-inflammatory, antimalarial, antidiabetic, and antibacterial activities [43,44,45,46,47,48,49,50].

Our study group has previously reported the neuroprotective potential of the synthesized curcumin analogues **C1**–**C3**, which might be used as neuroprotective agents in Alzheimer’s disease [45]**.** Keeping in mind the aforesaid properties of curcumin and their synthetic derivatives, the curcumin analogues **C1**–**C3**, with substituents at position 4, were selected for the current study based on their strong biological response to investigate their antistress and antidepressant potentials.

## 2. Materials and Methods

### 2.1. Chemicals and Animals

**Figure 1:**
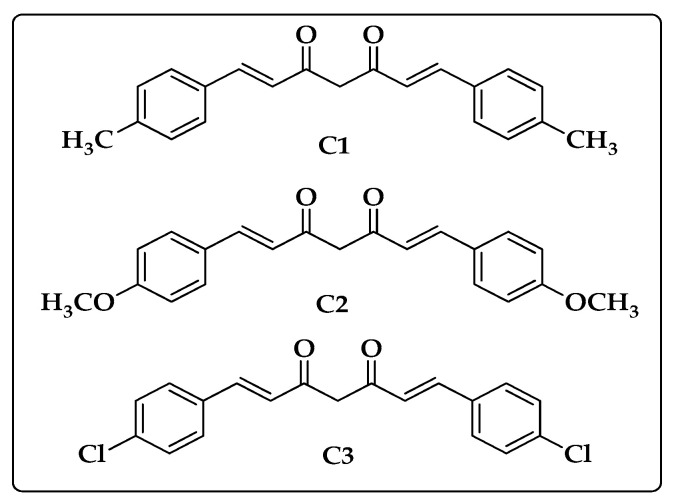
Chemical structures of synthetic curcumin analogues (**C1**–**C3**) used in this study.

The synthesized curcumin analogues **C1**–**C3** were used in this study, as previously described [45]. Figure 1 displays their chemical structures.

The tocopherol, DPPH, ABTS, and curcumin used in this study were from Sigma Aldrich (Merck, Darmstadt, Germany). Balb/C mice ranging between 19 and 23 g were obtained from the National Institute of Health (NIH), Islamabad. The animals were kept in an animal house with a 12 h light/dark cycle, diet and water ad libitum, a relative humidity of 55–65%, and a temperature of 25 ± 2 °C. The animals were exposed to laboratory conditions for two weeks before the experiments. The current study was done with approval from the “Departmental Ethical Committee” (notification number: SBBU/IEC-20-02). Mice were cared for according to the “Scientific Procedure Issue-I” animal bylaws of the University of Malakand in 2008.

### 2.2. In Vitro Antioxidant Activity

The antioxidant potentials of the curcumin analogues **C1**–**C3** were determined using DPPH and ABTS free radicals according to Brand-Williams’ 1995 procedure [51]. In the DPPH assay, the reference curcumin, tocopherol and curcumin analogues **C1**–**C3** at concentrations of 31.25–1000 µg/mL were mixed with the DPPH solution. The spectrophotometer, Shimadzu UV-1800, Kyoto, Japan, was run at 517 nm and the absorbance was noted. Similarly, in the ABTS assay, a 0.1 mL sample and standard solution at the same concentrations were treated with the ABTS, and at 734 nm the absorbance was recorded. Free radical-scavenging activities were measured for DPPH and ABTS. Finally, the IC_50_ values were calculated.

### 2.3. Acute Toxicity Study and Selection of Dose

The safety of curcumin analogues (**C1**–**C3**) was determined by examining their acute toxicity in mice for possible toxicological effects, determining that doses up to 150 mg/kg body weight were safe. In the in vivo models, 15 mg/kg of curcumin analogues was the optimal dosage [45].

### 2.4. Experimental Design and Animal Dosing

**Figure 2:**
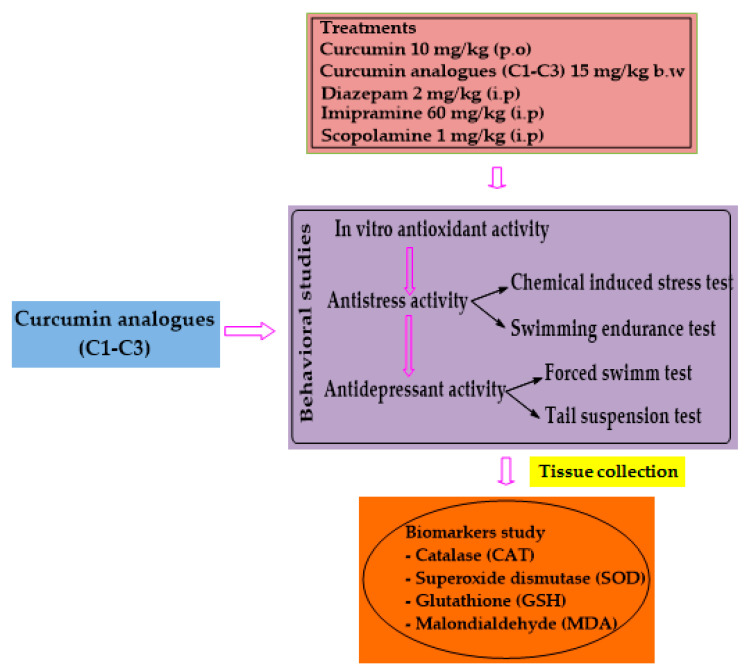
Sequential experimental outline of the activities used in this study.

The animals were categorized into different groups (n = 8), and received the following treatment doses: control group (2% Tween 80 in normal saline) 5 mL/kg (p.o), the stress control group in chemical-induced stress test received 0.1 mL (6% acetic acid), reference standards (curcumin 10 mg/kg p.o, diazepam 2 mg/kg i.p, imipramine 60 mg/kg p.o, donepezil 2 mg/kg b.w, p.o) and synthesized curcumin analogues **C1**–**C3** (15 mg/kg b.w (p.o)) in their respective activities were administered for 7 days. On the 7th day, the behavioral tests were performed. In the biomarkers study, on the 7th day, 60 min after the last dose of the tested compounds and standard drug, 1 mg/kg (i.p) scopolamine was administered. The experimental animals were immediately sacrificed for brain hippocampus tissue separation for biochemical analysis. The sequential experimental outline is presented in Figure 2.

### 2.5. Antistress Activity

The antistress effects of the curcumin analogues **C1**–**C3** were evaluated using a chemical-induced mice stress model involving the induction of stress via a chemical (acetic acid) and a swimming endurance test involving the induction of stress via swimming procedures.

#### 2.5.1. Chemical-Induced Stress

The curcumin analogues (**C1**–**C3**) were evaluated for antistress potential using a chemical-induced stress mice model. Animals were categorized randomly into groups, each carrying 8 animals, and 2% Tween 80 was administered to the control group. Following testing in 1–150 mg/kg body weight doses, preliminary analysis revealed that the 15 mg/kg dose was an effective dose for in vivo study. The curcumin analogues were administered to each group at a dose of 15 mg/kg. The standard drug diazepam 2 mg/kg (i.p) and curcumin 10 mg/kg (p.o) were given to the standard control group and all groups were continuously administered for 7 days. On the 7th day, one hour after the samples were administered, the animals in each group were treated with 0.1 mL of acetic acid (6% *v*/*v*) i.p. Animals were observed continuously for 20 min and the number of writhes performed by each animal was recorded [2,52].

#### 2.5.2. Swimming Endurance Test

Physical stress was induced in mice using a swimming endurance test according to standard procedures reported by Hardin in 1968 [52,53] with slight modifications. Animals were categorized randomly into different groups, each having 8 animals. Saline (2% Tween 80) was administered to the control group. The curcumin analogues were administered in a 15 mg/kg dose to the treatment groups. The standard control group received diazepam 2 mg/kg (i.p) and curcumin was used as the reference standard. The animals were consistently treated for 7 days. Animals were freely allowed to swim on the 7th day, one hour after the last dose in a water tank. Animals were continuously observed for 30 min, and the immobility time was recorded.

### 2.6. Antidepressant Activity

The forced swim test (FST) and the tail suspension test (TST) were used to assess antidepressant effects. Numerous studies used different animal models for evaluating behavioral and antidepressant effects mice and rats using the FST and TST, which are considered reliable tools for evaluating the antidepressant effects of various compounds [40,42].

#### 2.6.1. Forced Swim Test

The forced swim test (FST) for the examination of antidepressant-like behavior was undertaken with minor modifications to the standard procedure reported by Porsolt et al., 1977 [54,55]. Animals were subjected to a 15 min pre-swim test using a clear, cylindrical-shaped apparatus of 25 cm in height and 10 cm in diameter, at a depth of 10 cm of water and a temperature of 24 ± 1 °C. Mice were reintroduced to the apparatus 24 h later under the same conditions as the pre-test for 6 min. The duration of each mouse’s immobility throughout the last four minutes of the test was recorded. Mice were considered immobile once they stopped struggling and floated motionless in water. Mice exhibited the minimal movements required to keep their heads above water.

#### 2.6.2. Tail Suspension Test

In this experiment, mice were hung by their tails using sticky tape, starting 1 cm from the end of their tails. The tail was fixed using a black plastic box with dimensions of 20 × 20 × 45 cm and a front opening. The mice were suspended from their tails 40 cm above the floor. Immobility is defined as the absence of any movements other than those required for breathing. The immobility duration of each animal suspended from its tail in the air was monitored for a period of 5 min according to the standard procedure reported by Steru et al. 1985 [56].

### 2.7. Assessment of Biochemical Parameters and Biomarker Study

A scopolamine model was used to explore the role of biomarkers in oxidative stress. The animals were continuously treated for 14 days, and on the 14th day, scopolamine was administered by injection at 1 mg/kg, i.p to different groups, including control and sample-treated groups. Immediately after the completion of the scopolamine-induced stress behavioral study, the mice were subjected to cervical dislocation, providing a safe and painless death. The brain was isolated and homogenized to derive the hippocampus and then chilled in phosphate buffer saline before the biomarker study. The antioxidant enzyme levels, including catalase (CAT), superoxide dismutase (SOD), glutathione (GSH), and malondialdehyde (MDA), were measured according to standard procedures [2].

#### 2.7.1. Catalase (CAT) Activity

The catalase activity in the hippocampus was determined according to the standard procedure reported by Sinha in 1972 [57], with a slight modification by mixing 0.01M of 1 mL phosphate buffer having a pH 7 with 0.1 mL tissue homogenate and 2 M H_2_O_2_. Potassium dichromate 5% with acetic acid in 1:3 was added along with 2 mL dichromate acetic acid to the reaction mixture, and at 620 nm the absorbance was recorded. The activity of catalase was presented as the µM of H_2_O_2_ decomposed protein/min/mg.

#### 2.7.2. Superoxide Dismutase (SOD) Activity

The superoxide dismutase level was measured according to the standard procedure reported by Kakkar et al. in 1984 [58], with a little modification in which (0.5 mL) brain homogenate was diluted in 1 mL distilled water and mixed with 1.5 mL chloroform and 2.5 mL ethanol. The mixture was centrifuged at 4 °C for 1 min. Sodium pyrophosphate buffer 1.2 mL with a pH of 8.4, 0.025 M, was shaken with brain supernatant, and then 0.1 mL of 186 µMPMS, 30 µMNBT (0.3 mL) was added, and finally NADH 0.2 mL (780 µM) and 3 mL distilled water. The mixture was then incubated at 30 °C for 90 s, and then the reaction was stopped by adding 1 mL acetic acid. This was then stirred, and n-butanol was added and stirred again. The layer of butanol was removed, and against the blank butanol, the absorbance at 560 nm was recorded. The SOD quantity was measured as unit/mg of protein.

#### 2.7.3. Measurement of Glutathione (GSH) Activity

The glutathione (GSH) activity of curcumin analogues was determined according to the standard procedure reported by Moron et al. in 1979 [59]. In this method, 0.4 mL of brain homogenate was added to 20% 0.4 mL of TCA, and this was centrifuged at 4 °C for 20 min at 10,000× *g*. In total, 0.25 mL of supernatant was mixed with 0.2 M, pH 8.0 phosphate buffer and 2 mL 0.6 M DTNB to get 3 mL of the final volume. The absorbance at 412 nm was measured and the GSH concentration is presented as µM/mg of protein.

#### 2.7.4. Measurement of Malondialdehyde (MDA) Level

The MDA level in the hippocampus was determined according to the reported standard procedure, with little modification for the assessment of the antistress activity of the curcumin analogues (**C1**–**C3**) reported by Ohkawa et al. in 1979 [60]. Brain homogenate, 100 µL, was mixed with TBA 1.5 mL (0.8% *w*/*v*), sodium dodecyl sulfate 200 µL (8% *w*/*v*), and acetic acid 1.5 mL (20% *v*/*v*). The mixture was heated for 1 h at 90 °C and mixed with n-butanol 5 mL at room temperature after cooling. The organic layer was collected after the centrifugation of the mixture for 10 min at 976× *g*. Finally, absorbance was recorded at 532 nm.

### 2.8. Statistical Analysis

The results of this study were statistically analyzed by one-way ANOVA, followed by Dunnet’s multiple comparison tests, which were applied to the data set using Graph-Pad Prism (version 5.01) and are expressed in mean ± SEM.

## 3. Results

### 3.1. In Vitro Antioxidant Activity

The curcumin analogues (**C1**–**C3**) produced a significant antioxidant response in comparison to curcumin and the standard drug (Table 1). The synthesized symmetrical curcumin analogs, including methoxy-substituted **C2**, showed a significant free radical-scavenging potential in the DPPH assay, having an IC_50_ value of 45.18 ± 2.17 µg/mL, followed by chloro-substituted **C3,** with an IC_50_ value of 85.23 ± 1.96 µg/mL, and methyl-substituted **C1** having an IC_50_ value of 190.37 ± 1.83 µg/mL in comparison to curcumin and the standard drug tocopherol. Similarly, these analogues demonstrated promising antioxidant responses against ABTS free radicals, with methoxy-substituted analogue **C2** outperforming the standard drug. According to the results of this experiment, methoxy-substituted symmetrical curcumin analogue C2 exhibited greater free radical scavenging activity.

### 3.2. Acute Toxicity

The synthesized curcumin analogues (**C1**–**C3**) were found safe up to 150 mg/kg body weight after testing in mice for possible toxicological effects.

### 3.3. Chemical-Induced Stress

The synthesized curcumin analogues (**C1**–**C3**) significantly reduced the acetic acid-induced stress in mice by reducing the number of writhes over a period of 20 min after injecting acetic acid, in comparison to the stress control group (Table 2). Among the curcumin analogues, **C2** showed the maximum antistress response, and indicated a significant reduction in the number of writhes—11.18 ± 2.87, 80.17% (*p* < 0.001), n = 8—which indicated protection against the acetic acid-induced stress. The curcumin analogue **C3** also significantly reduced the number of writhes to 15.34 ± 1.34, 72.79% (*p* < 0.001), n = 8, while **C1** reduced the number of writhes to 27.15 ± 2.54, 51.85%, (*p* < 0.05), n = 8, in comparison to the stress control group, and showed a moderate antistress response. Curcumin significantly reduced the acetic acid-induced stress by 7.32 ± 1.61, 87.01% (*p* < 0.001). Diazepam also significantly reduced the number of writhes by 6.18 ± 1.87, 89.04% (*p* < 0.001), n = 8, and showed the maximum antistress response in mice by reducing the acetic acid-induced stress.

### 3.4. Swimming Endurance Test

When mice were subjected to a swimming endurance test, the curcumin analogues (**C1**–**C3**) produced promising results (Table 3). As compared to the stress control group, mice treated consistently for 7 days with 15 mg/kg of synthesized curcumin analogues showed a considerable increase in swimming time and a significant reduction in immobility time. Increased swimming duration and reduced immobility time served as antistress response parameters for curcumin analogues. Among the curcumin analogues, **C2,** with a methoxy substituent, showed the greatest antistress response of 6.75 ± 2.28 min (*p* < 0.001), n = 8, by significantly reducing the immobility time and increasing the swimming duration compared to the stress control group. By reducing the immobility time and increasing the swimming time, the chloro-substituent curcumin analogue **C3** indicated a promising antistress response of 8.61 ± 1.28 min (*p* < 0.01), while the methyl-substituted curcumin analogue **C1** demonstrated a poor (11.13 ± 1.53 min (*p* ˃ 0.05)) antistress response compared to the stress control group. Curcumin significantly reduced (6.91 ± 2.18 (*p* < 0.001)) the immobility time and showed a maximum antistress response in comparison to the stress control group. The standard drug diazepam also revealed a promising antistress response by significantly reducing immobility time and increasing the swimming duration by 5.26 ± 2.19 (*p* < 0.001), in comparison to the stress control group. This study indicated that curcumin analogues **C2** and **C3** offer antistress responses that are equally as potent as curcumin.

### 3.5. Antidepressant Activity

#### 3.5.1. Forced Swim Test (FST)

The results of the forced swim test (FST) are presented in Table 4. The antidepressant effects of the curcumin analogues (**C1**–**C3**) were investigated in the form of the active behavior of the mice by a decrease in immobility. The immobility time was significantly reduced upon administration of curcumin analogue **C2** (88.1 ± 3.19 s (*p* < 0.001)), followed by **C3** (106.17 ± 4.31 s (*p* < 0.01)) and then **C1** (139.16 ± 3.41 s (*p* < 0.05), n = 8) in comparison to the control group. The standard drug imipramine and curcumin have also shown a significant reduction in immobility time of 68.7 ± 3.72 s (*p* < 0.001), and 90.32 ± 1.18 (*p* < 0.001), respectively, in comparison to the vehicle control group. It has been revealed from the results that, among the curcumin analogues, the methoxy-substituent **C2** was found to be more potent than curcumin by reducing the immobility time and yielding a prominent antidepressant response.

#### 3.5.2. Tail Suspension Test (TST)

In Table 4, the antidepressant effects of the curcumin analogues (**C1**–**C3**) in terms of a decrease in the immobility time in the FST are presented. The standard drug imipramine significantly reduced (62.18 ± 1.83 s (*p* < 0.001)) the immobility time in comparison to the vehicle control group. The methoxy-substituent curcumin analogue **C2** significantly reduced the immobility time to 103.61 ± 2.51 s (*p* < 0.01), and showed the maximum antidepressant response, followed by the chloro-substituent curcumin analogue **C3** at 127.25 ± 1.38 s (*p* < 0.05), n = 8, at 15 mg/kg in comparison to the vehicle control group, while **C1** showed no promising antidepressant response among the curcumin analogues in comparison to the vehicle treatment group. Similarly, the natural curcumin has also shown prominent antidepressant effects by significantly reducing the immobility time to 69.81 ± 1.37, (*p* < 0.001), n = 8.

### 3.6. Scopolamine-Induced Oxidative Stress

To investigate the possible involvement of the antioxidant system in the attenuation of oxidative stress by curcumin analogues (**C1**–**C3**), the hippocampal-based scopolamine-induced stress model was used. In this study, scopolamine 1 mg/kg (i.p) was administered, which induced oxidative stress by drastically reducing endogenous antioxidant enzymes such as catalase, superoxide dismutase, and glutathione, and increasing the level of lipid peroxidation. The pretreatment with curcumin, curcumin analogues, and the standard drug consecutively for 14 days reversed the oxidative stress, increased the activity of antioxidant enzymes, and decreased the amount of lipid peroxidation.

#### Assessment of Biochemical Parameters and Biomarker Study

**Figure 3:**
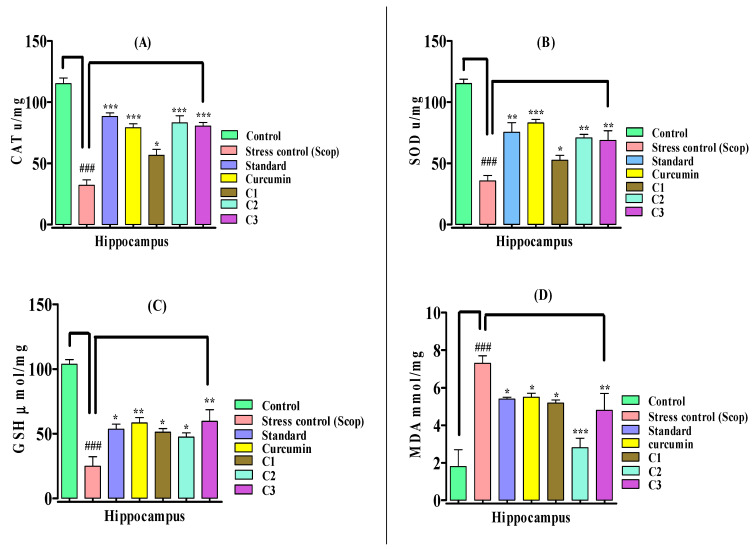
Effect of curcumin analogues (**C1**–**C3**) on the antioxidants system in scopolamine-induced mice. (**A**) CAT (catalase) activity, (**B**) SOD (superoxide dismutase) activity, (**C**) GSH (glutathione) activity, (**D**) MDA (malondialdehyde) level in the hippocampus of the mouse brain was measured. The standard drug donepezil and curcumin were used as reference standards. Mean ± SEM, n = 8, statistically significant data are shown as *p* < 0.05 *, *p* < 0.01 ** and *p* < 0.001 *** vs. stress control group. ^###^ *p* < 0.001 vs. control.

The biomarker study was conducted immediately after the in vivo scopolamine-induced oxidative stress behavioral model for the investigation of the antioxidant effects of curcumin analogues. In this study, scopolamine 1 mg/kg (i.p) administration caused oxidative stress via a significant reduction (*p* < 0.001) in the catalase level as compared to the normal control group mice, which was significantly elevated by the standard (*p* < 0.001), curcumin (*p* < 0.001), **C2** (*p* < 0.001), **C3** (*p* < 0.001), and **C1** (*p* < 0.05), n = 8, respectively, by protecting the brain from scopolamine-induced oxidative stress. The curcumin analogues **C2** and **C3** showed equal potency to curcumin by enhancing catalase activity (Figure 3A).

A significant reduction was observed in the superoxide dismutase activity when treated with scopolamine in comparison to the normal control group in the hippocampus region of the mouse brain that produces oxidative stress (Figure 3B). The treatments groups administered with curcumin analogues, **C2** (*p* < 0.01), **C3** (*p* < 0.01) and **C1** (*p* < 0.05), n = 8, significantly increased the superoxide dismutase (SOD) in comparison to the stress control group. In this experiment, curcumin was found to be more potent compared to curcumin analogues by significantly (*p* < 0.001) enhancing the SOD level. The SOD activity showed the protective effects of the curcmin and curcumin analogues against oxidative stress. A significant reduction (*p* < 0.001) in the glutathione (GSH) was observed among the groups treated with scopolamine, which caused severe oxidative stress in comparison to normal control (Figure 3C). The pretreatment with the tested compounds markedly enhanced the GSH level—**C3** (*p* < 0.01), **C1** (*p* < 0.05) **C2** (*p* < 0.05), n = 8—and showed moderate antioxidant activities in comparison to the scopolamine-induced stress groups. Curcumin also showed a promising response (*p* < 0.01) similar to the other tested compounds in comparison to the stress control group.

As indicated in Figure 3D, scopolamine administration raised the lipid peroxidation level in the hippocampus of mice, and caused a significant increase in MDA (*p* < 0.001) level, which produced oxidative stress in the scopolamine-treated groups in comparison to the normal control group. Pretreatment with curcumin analogues prevented the formation of lipid peroxidation resulting from oxidative stress in the hippocampus region of the mouse brain. The MDA level was significantly reduced with the methoxy-substituent curcumin analogue **C2** (*p* < 0.001) (showing higher antioxidant activity), the chloro-substituent curcumin analogue **C3** (*p* < 0.01) (showing moderate activity), and methyl-substituent curcumin analogue **C1** (*p* < 0.05) (indicating a weaker antioxidant response). The standard control group and curcumin also showed a weaker (*p* < 0.05) antioxidant response, slightly reducing the MDA level (*p* < 0.05), n = 8, in the hippocampus of the mouse brain. This study shows that curcumin analogues **C2** and **C3** were more potent than natural curcumin, and acted by attenuating oxidative stress in mice.

## 4. Discussion

Stress is the body’s physical and mental response to extreme circumstances in order to ensure survival [61]. Extremely stressful conditions have been linked to depression, immunosuppression, hypertension, and endocrine disorders [33]. Multiple factors, including psychological influences, as well as genetic and biological factors, are also involved in the initiation of depressive episodes [11]. Facial emotional expressions are among the contextual factors that can modulate brain activations, and help in the understanding of the manners of social cognition linked with numerous neurological and psychiatric disorders [12]. Numerous drugs are now used to treat depression and stress. Nevertheless, a number of drugs are used to manage stress and depression. However, these compounds are accompanied by several adverse effects and toxicity. Consequently, the present work has defined a concept of identifying a beneficial molecule for stress management by modifying the structure of curcumin and producing various analogues. It has been shown in research studies that compounds containing methoxy (-OCH3) and hydroxyl (-OH) moieties in their structures play a potential role in scavenging free radicals [62]. Similarly, a synthesized curcumin analogue with a methoxy-substituent showed higher free radical-scavenging activity. Curcumin analogues with methoxy-substituent have also been shown in the literature to have promising antioxidant and anti-amyloid protein aggregate activities [63,64], and anti-β amyloid protein aggregate activity [65]. The antioxidant profile of these compounds can be used for various brain ailments associated with oxidative stress.

Stress was induced via the administration of acetic acid in the chemical-induced stress mice, indicating the hyperalgesic phenomenon involving the nociception path, as indicated by an increase in the number of writhes following the induction of stress. “Writhing is defined as a stretch, tension to one side, extension of the hind legs, or contraction of the abdomen so that the abdomen of the mice touches the floor or turning of the trunk (twist)” [66]. This study demonstrated a significant reduction in the number of writhes, and showed that curcumin and curcumin analogues inhibited pain by providing an antistress response, which is consistent with previous studies [67]. Stress was induced in the swimming endurance test physically, by forcing the mice to swim in a constrained area of water, preventing them from escaping. The agents with antistress activity increased the swimming endurance, and decreasing immobility times were reported [2]. In this study, using a swimming endurance model, the curcumin analogues at 15 mg/kg showed a marked reduction in the immobility time, and revealed antistress potential. The forced swim test and tail suspension test are the most widely used mice models for the assessment of the antidepressant effects of pharmacological substances [68]. The immobility time was significantly reduced by the curcumin and curcumin analogues in both FST and TST, which exhibited antidepressant potential. The depressed patients showed disordered oxidative stress, related to abnormal oxidative stress marker levels [21]. This study suggests that treatment with our curcumin analogues revered and fixed the abnormal oxidative stress markers.

The nervous system, under stressful circumstances, is immensely prone to increased levels of MDA as a result of high oxygen tension [2]. Scopolamine administration induced serious oxidative stress, which was demonstrated by the disordered oxidative stress markers, including antioxidants such as CAT, SOD, GSH, and MDA, and is also associated with memory loss [69]. Curcumin and curcumin analogues reduced the scopolamine-induced oxidative stress in the hippocampus of the mouse brain. Numerous studies have shown that curcumin considerably reduces oxidative stress and has significant antioxidant effects [39,70]. According to research, scopolamine induces oxidative stress in the brain tissue of mice by increasing lipid peroxidation and decreasing the activity of antioxidant enzymes [71,72]. The current study’s results are consistent with this hypothesis, and reveal higher levels of lipid peroxidation and decreased activities of antioxidant enzymes in the scopolamine-treated groups. The curcumin analogues significantly increased CAT and SOD activity, and moderately increased GSH activity; they also showed a higher antioxidant response by decreasing the MDA level. These compounds were found to be more potent in comparison to curcumin. These results are parallel with the previous findings [39]. The current study showed a promising reduction in the lipid peroxidase level and an enhanced GSH activity. This is consistent with previous findings [39].

The curcumin analogues significantly reduced the lipid peroxidation level, which shows that their antioxidant effects are mediated by reductions in oxidative stress. The use of curcumin analogues improved cell survival and neurogenesis [73]. Depression is a direct result of changes in hippocampal neurogenesis. This study suggests that antidepressant-like effects are mediated by an increase in hippocampal neurogenesis [74,75,76,77]. The investigation of hippocampal biomarkers in scopolamine-induced stress mouse models may aid in understanding the crucial function of endogenous antioxidants in oxidative stress and depression-related neurological disorders.

### Limitations

This study offers initial findings, which are restricted to preliminary antistress and antidepressant studies without the use of advanced procedures, such as Western blot analysis, PCR, MTT, etc. By investigating the exact mechanism of action, it is suggested that more research is needed to determine the synthetic curcumin molecule that will be useful for the treatment of oxidative stress and stress-related psychological disorders.

## 5. Conclusions

In conclusion, these curcumin analogues could protect the brain from the damage of reactive oxygen species by reducing oxidative stress and potentiating antioxidant systems in the hippocampus, and they may be valuable therapeutic molecules for relieving depression associated with oxidative and psychological stress. The curcumin analogues with various substituents are comparable in their antistress and antidepressant responses to natural curcumin. This work offers initial findings, and further exploration is needed to investigate the mechanistic insights and molecular targets.

## Figures and Tables

**Table 1:** In vitro antioxidant activity of synthesized curcumin analogs (**C1**–**C3**).

Compound	DPPH (IC_50_ µg/mL)	ABTS (IC_50_ µg/mL)
C1	190.37 ± 1.83	220.13 ± 2.35
C2	45.18 ± 2.17	62.31 ± 1.61
C3	85.23 ± 1.96	123.53 ± 1.18
Curcumin	18.13 ± 1.08	23.72 ± 1.14
Tocopherol	8.28 ± 1.42	12.35 ± 1.89

Mean ± SEM, (n = 3) and values that were significantly different were compared to positive controls.

**Table 2:** Effects of curcumin analogues (**C1**–**C3**) on chemical-induced test.

Group	Number of Writhes	Antistress Response (%)
Control	0.83 ± 0.98	-
Stress control	51.17 ± 2.18 ^†††^	9.25
Standard control	6.18 ± 1.87 ***	89.04
Curcumin	7.32 ± 1.61 ***	87.01
C1	27.15 ± 2.54 *	51.85
C2	11.18 ± 2.87 ***	80.17
C3	15.34 ± 1.34 ***	72.79

Mean ± SEM, *n* = 8, *p* < 0.001 ***, and *p* < 0.05 * vs. stress control, ^†††^ *p* < 0.001 vs. control group.

**Table 3:** Effects of synthesized curcumin analogues (**C1**–**C3**) on swimming endurance test.

Group	Immobility Time (min)
Control	6.39 ± 1.26
Stress control	15.19 ± 1.71 ^†††^
Curcumin	6.91 ± 2.18 ***
C1	11.13 ± 1.53 ^ns^
C2	6.75 ± 2.28 ***
C3	8.61 ± 1.28 **
Standard	5.26 ± 2.19 ***

Mean ± SEM, n = 8, *p* < 0.001 ***, and *p* < 0.01 **, *p* ˃ 0.05 ^ns^ vs. stress control. ^†††^ *p* < 0.001 vs. control.

**Table 4:** Effects of curcumin analogues (**C1**–**C3**) on immobility time in mice in the FST and TST.

Group	Immobility Time (s) FST	Immobility Time (s) TST
Control	182.30 ± 2.91	176.51 ± 1.62
Curcumin	90.32 ± 1.18 ***	69.81 ± 1.37 ***
Standard control	68.71 ± 1.72 ***	62.18 ± 1.83 ***
C1	139.16 ± 1.86 *	164.29 ± 1.15 ^ns^
C2	88.17 ± 1.49 ***	103.61 ± 2.51 **
C3	106.17 ± 1.93 **	127.25 ± 1.38 *

Mean ± SEM, n = 8; statistically significant data are shown as *p* ˃ 0.05 ^ns^, *p* < 0.05 *, *p* < 0.01 ** and *p* < 0.001 *** vs. vehicle treated groups. Animals were treated with curcumin analogues (**C1**–**C3**) 15 mg/kg, curcumin 10 mg/kg, vehicle and imipramine (standard) 60 mg/kg. One-way ANOVA was applied, followed by Dunnett’s test.

## Data Availability

All data contained within this article.

## References

[1] Hurley L.L., Akinfiresoye L., Nwulia E., Kamiya A., Kulkarni A., Tizabi Y. (2013). Antidepressant-like effects of curcumin in WKY rat model of depression is associated with an increase in hippocampal BDNF. Brain Behav. Res..

[2] Ghias M., Wadood S., Shah A., Al-joufi F.A., Shoaib M., Muhammad S., Shah M., Ahmed M.N., Zahoor M. (2022). In Vivo Antistress Effects of Synthetic Flavonoids in Mice: Behavioral and Biochemical Approach. Molecules.

[3] Bach F., Mills E., Cartledge P., Roberts H. (2021). Hyperactivity disorder. Biomedicines.

[4] Schneiderman N., Ironson G., Siegel S.D. (2005). Stress and health: Psychological, behavioral, and biological determinants. Annu. Rev. Clin. Psychol..

[5] Hammen C. (2005). Stress and depression. Annu. Rev. Clin. Psychol..

[6] Tanaka M., Tóth F., Polyák H., Szabó Á., Mándi Y., Vécsei L. (2021). Immune Influencers in Action: Metabolites and Enzymes of the Tryptophan-Kynurenine Metabolic Pathway. Biomedicines.

[7] Tanaka M., Vécsei L. (2021). Monitoring the kynurenine system: Concentrations, ratios or what else?. Adv. Clin. Exp. Med..

[8] Kim I., Lee J., Park S. (2022). The Relationship between Stress, Inflammation, and Depression. Biomedicines.

[9] Hestad K., Alexander J., Rootwelt H. (2022). The Role of Tryptophan Dysmetabolism and Quinolinic Acid in Depressive and Neurodegenerative Diseases. Biomedicines.

[10] Hepsomali P., Coxon C. (2022). Inflammation and diet: Focus on mental and cognitive health. Adv. Clin. Exp. Med..

[11] Pignataro P., Dicarlo M., Zerlotin R., Storlino G., Oranger A., Sanesi L., Lovero R., Buccoliero C., Mori G., Colaianni G. (2022). Antidepressant Effect of Intermittent Long-Term Systemic Administration of Irisin in Mice. Int. J. Mol. Sci..

[12] Battaglia S., Fabius J.H., Moravkova K., Fracasso A., Borgomaneri S. (2022). The Neurobiological Correlates of Gaze Perception in Healthy Individuals and Neurologic Patients. Biomedicines.

[13] Simone Battaglia J.F.T. (2022). Functional interplay between central and autonomic nervous systems in human fear conditioning. Trends Neurosci..

[14] Somani A., Singh A.K., Gupta B., Nagarkoti S., Dalal P.K., Dikshit M. (2022). Oxidative and Nitrosative Stress in Major Depressive Disorder: A Case Control Study. Brain Sci..

[15] Sarandol A., Sarandol E., Eker S.S., Erdinc S., Vatansever E. (2007). Major depressive disorder is accompanied with oxidative stress: Short-term antidepressant treatment does not alter oxidative—Antioxidative systems. Hum. Psychopharmacol..

[16] Cumurcu B.E., Ozyurt H., Etikan I., Demir S., Karlidag R. (2009). Total antioxidant capacity and total oxidant status in patients with major depression: Impact of antidepressant treatment. Psychiatry Clin. Neurosci..

[17] Ozan V., Sarandol E., Kirhan E., Ozkaya G., Kirli S. (2011). Progress in Neuro-Psychopharmacology & Biological Psychiatry Effects of long-term antidepressant treatment on oxidative status in major depressive disorder: A 24-week follow-up study. Prog. Neuropsychopharmacol. Biol. Psychiatry.

[18] Michel T.M., Pülschen D., Thome J. (2012). The Role of Oxidative Stress in Depressive Disorders. Curr. Pharm. Des..

[19] Bhatt S., Nagappa A.N., Patil C.R. (2020). Role of oxidative stress in depression. Drug Discov. Today.

[20] Rahman S.U., Ali T., Hao Q., He K., Li W., Ullah N., Zhang Z., Jiang Y., Li S. (2021). Xanthohumol Attenuates Lipopolysaccharide-Induced Depressive Like Behavior in Mice: Involvement of NF-κB/Nrf2 Signaling Pathways. Neurochem. Res..

[21] Peng Y., Chang X., Lang M. (2021). Iron homeostasis disorder and alzheimer’s disease. Int. J. Mol. Sci..

[22] Moneim A.E.A. (2015). Oxidant/Antioxidant Imbalance and the Risk of Alzheimer ’ s Disease. Curr. Alzheimer Res..

[23] Liu T., Zhong S., Liao X., Chen J., He T., Lai S. (2015). A Meta-Analysis of Oxidative Stress Markers in Depression. PLoS ONE.

[24] Kalogeris T., Bao Y., Korthuis R.J. (2014). Mitochondrial reactive oxygen species: A double edged sword in ischemia/reperfusion vs. preconditioning. Redox Biol..

[25] Ashutosh Bajpai A.K.V. (2014). Oxidative Stress and Major Depression. J. Clin. Diagn. Res..

[26] Vanzella C., Neves J.D., Vizuete A.F., Aristimunha D., Kolling J., Longoni A., Gonçalves C.A.S., Wyse A.T.S., Netto C.A. (2017). Treadmill running prevents age-related memory deficit and alters neurotrophic factors and oxidative damage in the hippocampus of Wistar rats. Behav. Brain Res..

[27] Borghans B. (2015). Animal models for posttraumatic stress disorder: An overview of what is used in research. World J. Psychiatry.

[28] Vilmosh N., Delev D., Kostadinov I., Zlatanova H., Kotetarova M., Kandilarov I., Kostadinova I. (2022). Anxiolytic Effect of Satureja montana Dry Extract and its Active Compounds Rosmarinic Acid and Carvacrol in Acute Stress Experimental Model. J. Integr. Neurosci..

[29] Shoaib M., Shah S.W.A., Ali N., Shah I., Naveed Umar M., Shafiullah, Ayaz M., Tahir M.N., Akhtar S. (2015). In vitro enzyme inhibition potentials and antioxidant activity of synthetic flavone derivatives. J. Chem..

[30] Ighodaro O.M., Akinloye O.A. (2018). First line defence antioxidants-superoxide dismutase (SOD), catalase (CAT) and glutathione peroxidase (GPX): Their fundamental role in the entire antioxidant defence grid. Alex. J. Med..

[31] Mošovská S., Petáková P. (2016). Antioxidant properties of curcuminoids isolated from *Curcuma longa* L.. Acta Chim. Slovaca.

[32] Kothiyal P., Ratan P. (2011). Kothiyal and Ratan Antistress Effect of Fagopyrum esculentum in Rats subjected to Forced Swimming Endurance Test. Kothiyal and Ratan. Pharmacologyonline.

[33] Tanvir E.M., Hossen S., Hossain F., Afroz R., Gan S.H., Khalil I., Karim N. (2017). Antioxidant Properties of Popular Turmeric ( Curcuma longa ) Varieties from Bangladesh. J. Food Qual..

[34] Desai S.K., Desai S.M., Navdeep S., Arya P., Pooja T. (2011). Antistress activity of Boerhaavia diffusa root extract and a polyherbal formulation containing Boerhaavia diffusa using cold restraint stress model. Int. J. Pharm. Pharm. Sci..

[35] Zintle Mbese V.K., Aderibigbe B.A. (2019). Curcumin and Its Derivatives as Potential Therapeutic Agents in Prostate, Colon and Breast Cancers. Molecules.

[36] Liang G., Yang S., Jiang L., Zhao Y., Shao L., Xiao J., Ye F., Li Y., Li X. (2008). Synthesis and anti-bacterial properties of mono-carbonyl analogues of curcumin. Chem. Pharm. Bull..

[37] Forms P. (2022). Curcumin: Biological Activities and Modern. Antibiotics.

[38] Ahmed T., Gilani A.H. (2009). Inhibitory effect of curcuminoids on acetylcholinesterase activity and attenuation of scopolamine-induced amnesia may explain medicinal use of turmeric in Alzheimer’s disease. Pharmacol. Biochem. Behav..

[39] Lee W., Loo C., Bebawy M., Luk F., Mason R.S. (2013). Curcumin and its Derivatives: Their Application in Neuropharmacology and Neuroscience in the 21 st Century. Curr. Neuropharmacol..

[40] Naqvi F., Haider S., Naqvi F., Saleem S., Perveen T., Batool Z. (2019). A comparative study showing greater effects of curcumin compared to donepezil on memory function in rats. Pak. J. Pharm. Sci..

[41] Seo H.J., Wang S.M., Han C., Lee S.J., Patkar A.A., Masand P.S., Pae C.U. (2015). Curcumin as a putative antidepressant. Expert Rev. Neurother..

[42] Kaufmann F.N., Gazal M., Bastos C.R., Kaster M.P., Ghisleni G. (2016). Curcumin in depressive disorders: An overview of potential mechanisms, preclinical and clinical findings. Eur. J. Pharmacol..

[43] Zhang Y., Li L., Zhang J. (2020). Curcumin in antidepressant treatments: An overview of potential mechanisms, pre-clinical/clinical trials and ongoing challenges. Basic Clin. Pharmacol. Toxicol..

[44] Warsi W., Sardjiman S., Riyanto S. (2018). Synthesis and Antioxidant Activity of Curcumin Analogues. J. Chem. Pharm. Res..

[45] Fadda A.A., Badria Æ.F.A., El-attar K.M. (2010). Synthesis and evaluation of curcumin analogues as cytotoxic agents. Med. Chem. Res..

[46] Hussain H., Ahmad S., Wadood S., Shah A., Ullah A., Ali N., Almehmadi M., Ahmad M., Ali A., Khalil K. (2022). Attenuation of Scopolamine-Induced Amnesia via Cholinergic Modulation in Mice by Synthetic Curcumin Analogs. Molecules.

[47] Hussain H., Ahmad S., Wadood S., Shah A., Ghias M., Ullah A., Rahman S.U., Kamal Z., Khan F.A., Khan N.M. (2021). Neuroprotective Potential of Synthetic Mono-Carbonyl Curcumin Analogs Assessed by Molecular Docking Studies. Molecules.

[48] Chainoglou E., Hadjipavlou-litina D. (2019). Curcumin analogues and derivatives with anti- proliferative and anti-inflammatory activity: Structural characteristics and molecular targets. Expert Opin. Drug Discov..

[49] Mishra S., Karmodiya K., Surolia A. (2008). Synthesis and exploration of novel curcumin analogues as anti-malarial agents. Bioorg. Med. Chem..

[50] Yuan X., Li H., Bai H., Su Z., Xiang Q., Wang C. (2014). Synthesis of novel curcumin analogues for inhibition of 11b-hydroxysteroid dehydrogenase type 1 with anti-diabetic properties. Eur. J. Med. Chem..

[51] Kim B.R., Park J., Jeong H.J., Kwon H., Park S., Lee I., Ryu Y.B., Lee W.S., Ram B., Park J. (2018). Design, synthesis, and evaluation of curcumin analogues as potential inhibitors of bacterial sialidase. J. Enzyme Inhib. Med. Chem..

[52] Brand-Williams W., Cuvelier M.E., Berset C. (1995). Use of a Free Radical Method to Evaluate Antioxidant Activity. Food Sci. Technol..

[53] Kulkarni M.P., Juvekar A.R. (2008). Attenuation of Acute and Chronic Restraint Stress- induced Perturbations in Experimental Animals by Nelumbo nucifera Gaertn. Indian J. Pharm. Sci..

[54] Hardin D.H. (1968). Reliability of Selected Swimming Endurance Tests for Laboratory Rats. Res. Q. Am. Assoc. Health Phys. Educ. Recreat..

[55] Porsolt R.D., Anton G., Blavet N., Jalfre M. (1978). Behavioural despair in rats: A new model sensitive to antidepressant treatments. Eur. J. Pharmacol..

[56] Abdelhalim A., Karim N., Chebib M., Aburjai T., Khan I., Johnston G.A.R., Hanrahan J.R. (2015). Antidepressant, anxiolytic and antinociceptive activities of constituents from rosmarinus officinalis. J. Pharm. Pharm. Sci..

[57] Steru L., Chermat R., Thierry B., Simon P. (1985). The tail suspension test: A new method for screening antidepressants in mice. Psychopharmacology.

[58] Sinha A.K. (1972). Colorimetric assay of catalase. Anal. Biochem..

[59] Kakkar P., Das B., Viswanathan P.N. (1984). A modified spectrophotometric assay of superoxide dismutase. Indian J. Biochem. Biophys..

[60] Moron M.S., Depierre J.W., Mannervik B. (1979). Levels of glutathione, glutathione reductase and glutathione S-transferase activities in rat lung and liver. Biochim. Biophys. Acta.

[61] Ohkawa H., Ohishi N., Yagi K. (1979). Assay for lipid peroxides in animal tissues by thiobarbituric acid reaction. Anal. Biochem..

[62] Shinohara H., Fukumitsu H., Seto A., Furukawa S. (2013). Medium-chain fatty acid-containing dietary oil alleviates the depression-like behaviour in mice exposed to stress due to chronic forced swimming. J. Funct. Foods.

[63] Zafar R., Ullah H., Zahoor M., Sadiq A. (2019). Isolation of bioactive compounds from Bergenia ciliata (haw.) Sternb rhizome and their antioxidant and anticholinesterase activities. BMC Complement. Altern. Med..

[64] Khushwant S. (2013). Bhullar Curcumin and Its Carbocyclic Analogs: Structure-Activity in Relation to Antioxidant and Selected Biological Properties. Molecules.

[65] Anand P., Thomas S.G., Kunnumakkara A.B., Sundaram C., Harikumar K.B., Sung B., Tharakan S.T., Misra K., Priyadarsini I.K., Rajasekharan K.N. (2008). Biological activities of curcumin and its analogues (Congeners) made by man and Mother Nature. Biochem. Pharmacol..

[66] Sajjad N., Wani A., Hassan S., Ali R., Hamid R., Akbar S., Ba G., Ea B. (2019). Interplay of antioxidants in Alzheimer’ s disease. J. Transl. Sci..

[67] Mishra D., Joshi S., Pilkhwal S., Bisht G. (2014). Chemical composition, analgesic and antimicrobial activity of Solidago canadensis essential oil from India Available online through Chemical composition, analgesic and antimicrobial activity of Solidago canadensis essential oil from India. J. Pharm. Res..

[68] Sheethal S., Ratheesh M., Jose S.P., Asha S., Krishnakumar I.M., Sandya S., Girishkumar B., Grace J. (2020). Anti-Ulcerative Effect of Curcumin-Galactomannoside Complex on Acetic Acid-Induced Experimental Model by Inhibiting Inflammation and Oxidative Stress. Inflammation.

[69] Debnath J., Prakash T., Karki R., Kotresha D., Sharma P. (2011). An Experimental Evaluation of Anti-stress Effects of Terminalia chebula. J. Physiol. Biomed. Sci..

[70] Jinyuan S. (2020). Effects of curcumin on physical fatigue and oxidative damage in forced swimming mice. E3S Web Conf..

[71] Nazir N., Zahoor M., Nisar M., Karim N., Latif A., Ahmad S., Uddin Z. (2020). Evaluation of neuroprotective and antiamnesic effects of elaeagnus umbellate thunb. On scopolamine-induced memory impairment in mice. BMC Complement. Med. Ther..

[72] Budzynska B., Boguszewska-czubara A., Kruk-slomka M., Skalicka-wozniak K., Michalak A., Musik I., Biala G. (2015). Effects of imperatorin on scopolamine-induced cognitive impairment and oxidative stress in mice. Psychopharmacology.

[73] Hatcher H. (2008). Curcumin: From ancient medicine to current clinical trials. Cell. Mol. Life Sci..

[74] Pathway B.T., Baek S.Y., Li F.Y., Kim D.H., Kim S.J., Kim M.R. (2020). Enteromorpha prolifera Extract Improves Memory in Scopolamine-Treated Mice via Downregulating Amyloid- β Expression and Upregulating. Antioxidants.

[75] Du C.N., Min A.Y., Kim H.J. (2015). Deer Bone Extract Prevents Against Scopolamine-Induced Memory Impairment in Mice. J. Med. Food.

[76] Nade V.S., Kawale L.A., Naik R.A., Yadav A.V. (2009). Adaptogenic effect of Morus alba on chronic footshock-induced stress in rats. Indian J. Pharmacol..

[77] First M., Gil-Ad I., Taler M., Tarasenko I., Novak N., Weizman A. (2011). The effects of fluoxetine treatment in a chronic mild stress rat model on depression-related behavior, brain neurotrophins and ERK expression. J. Mol. Neurosci..

